# Altered oncomodules underlie chromatin regulatory factors driver mutations

**DOI:** 10.18632/oncotarget.8752

**Published:** 2016-04-15

**Authors:** Joan Frigola, Ane Iturbide, Nuria Lopez-Bigas, Sandra Peiro, Abel Gonzalez-Perez

**Affiliations:** ^1^ Research Program on Biomedical Informatics, IMIM Hospital del Mar Medical Research Institute and Universitat Pompeu Fabra, 08003 Barcelona, Catalonia, Spain; ^2^ Programa de Recerca en Càncer, Institut Hospital del Mar d'Investigacions Mèdiques (IMIM), 08003 Barcelona, Spain; ^3^ Institució Catalana de Recerca i Estudis Avançats (ICREA), 08010 Barcelona, Spain

**Keywords:** chromatin regulatory factors, CRFs oncogenic modules, indirect targeted therapeutic strategies, CRFs Oncomodules Discovery, oncogenic modules scoring system

## Abstract

Chromatin regulatory factors (CRFs), are known to be involved in tumorigenesis in several cancer types. Nevertheless, the molecular mechanisms through which driver alterations of CRFs cause tumorigenesis remain unknown. Here, we developed a CRFs Oncomodules Discovery approach, which mines several sources of cancer genomics and perturbaomics data. The approach prioritizes sets of genes significantly miss-regulated in primary tumors (oncomodules) bearing mutations of driver CRFs. We applied the approach to eleven TCGA tumor cohorts and uncovered oncomodules potentially associated to mutations of five driver CRFs in three cancer types. Our results revealed, for example, the potential involvement of the mTOR pathway in the development of tumors with loss-of-function mutations of *MLL2* in head and neck squamous cell carcinomas. The experimental validation that *MLL2* loss-of-function increases the sensitivity of cancer cell lines to mTOR inhibition lends further support to the validity of our approach. The potential oncogenic modules detected by our approach may guide experiments proposing ways to indirectly target driver mutations of CRFs.

## INTRODUCTION

In recent years, catalogs of mutational cancer driver genes from large sequencing datasets have been identified [1, 2]. Although most of such mutational drivers are involved in biological processes traditionally associated with cancer, such as apoptosis or cell proliferation [3, 4], an important fraction [1] is related to cellular regulatory functions, including the regulation of chromatin structure. Chromatin remodeling is crucial to the regulation of gene expression. Three main biochemical mechanisms compose chromatin remodeling –covalent histone modifications, core histone replacement and ATP-dependent chromatin remodeling [5]. Proteins that carry out these three processes are generically referred to as chromatin regulatory factors (CRFs), and their involvement in tumorigenesis is now well established [6]. We recently showed that i) drivers are overrepresented within CRFs; ii) CRF complexes –such as SWI/SNF [7]– rather than individual genes driver tumorigenesis; and iii) the importance of CRFs in tumorigenesis varies amongst cancer types [8]. However, in most cases the actual mechanism through which mutations in driver CRFs lead to tumorigenesis is unclear. In this work, we start with the catalog of mutational driver CRFs in a cohort of almost 7.000 tumors representing 29 cancer types, extending the aforementioned previous analysis. We then hypothesize that changes in the expression of key groups of genes mediate the tumorigenic effect of mutational driver CRFs. To test this hypothesis, we develop a simple three-step bioinformatics approach –the CRFs Oncomodules Discovery Approach, or CRFs-ODA. We first culled from TCGA a dataset of 3583 tumor samples from 11 cancer types for which both mutation and expression data are available [9]. We then systematically detected genes whose expression changes significantly in coherence with mutations in individual driver CRFs. We call the groups of functionally related genes (i.e. those in biochemical pathways, gene ontology terms, or under the regulation of a transcription factor, etc) significantly enriched for the differentially expressed genes in the previous analysis, oncomodules. Finally, we ranked these oncomodules according to prior knowledge on tumorigenesis and information from several high throughput cancer genomics and perturbaomics datasets [10, 11]. It is thus possible to construct hypotheses linking the somatic mutations in the driver CRF and the emergence of cancer based on the top-ranking oncomodules. Furthermore, they provide clues on possible therapeutic strategies to indirectly target tumors bearing mutated CRFs.

## RESULTS

### CRFs as mutational drivers across tumor types

We started with a list of 459 mutational drivers derived from the combination of three widely-used bioinformatics methods [12–14] to a cohort of almost 7,000 tumors of 29 cancer types [1]. We intersected this list with a manually curated set of 183 CRFs [8], resulting in 37 cancer driver CRFs, 24 of which are included in the Cancer Gene Census, CGC [15]. (The refined approach employed to detect the drivers [16] and the increase in the number of tumor samples in the cohort analyzed constitute the main improvement of this catalog of driver CRFs over the aforementioned study.) CRFs as a group are significantly enriched for drivers, as 25.3% of known CRFs are drivers, which represent only 1.9% of non-CRF human genes (Fisher's test p-value: 2.2×10^−16^). Three quarters of these CRFs (77.7%) are predicted to be loss-of-function (LoF) [17]. On the other hand, only 48% of all drivers are predicted LoF.

**Figure 1:**
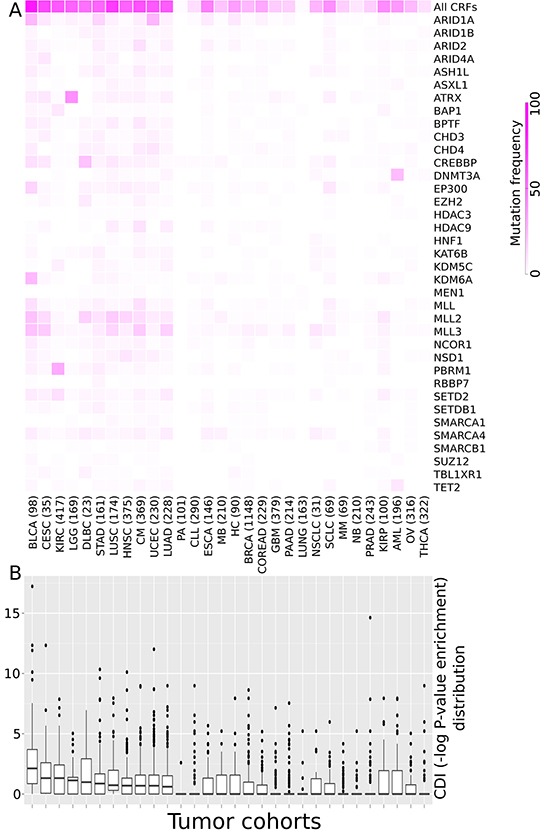
CRFs and their relative importance as drivers across tumor types **A.** Heatmap illustrating the frequency of samples with mutations of each known driver CRF relative to the total number of samples of 30 cohorts of tumors. (A cohort of lung tumors of unspecified histology was added to those of the 29 tumor types analyzed in our aforementioned work. Note that because it does not represent a new tumor type, the cohort under study still represents tumors from 29 cancer types.) **B.** The boxplots show the distribution of the enrichment for driver mutations of CRFs across all samples of each cohort (CDI, see text for details). The enrichment for driver mutations of CRFs in each sample was computed as the minus logarithm of the p-value of a Fisher's exact test of the overrepresentation of mutations in driver CRFs in each sample through a contingency table. The tumor cohorts in both panels are sorted by decreasing CDI median value. Tumor type acronyms: BLCA: Bladder carcinomas; CESC: Cervical squamous cell carcinoma and endocervical adenocarcinoma; KIRC: Renal clear cell carcinoma; LGG: Lower grade glioma; DLBC: Difuse large B-cell linfoma; STAD: Stomach adenocarcinoma; LUSC: Lung squamous cell carcinoma; HNSC: Head and neck squamous cell carcinoma; CM: Cutaneous melanoma; UCEC: Uterine endometrioid carcinoma; LUAD: Lung adenocarcinoma; PA: Pilocytic astrocytoma; CLL: Chronic lymphocytic leukemia; ESCA: Esophageal carcinoma; MB: Medulloblastoma; HC: Hepatocellular carcinoma; BRCA: Breast carcinoma; COREAD: Colorectal adenocarcinoma; GBM: Glioblastoma multiforme; PAAD: Pancreatic adenocarcinoma; Lung: Lung cancer (histology unspecified); NSCLC: Non-small cell lung cancer; SCLC: Small cell lung cancer; MM: Multiple myeloma; NB: Neuroblastoma; PRAD: Prostate adenocarcinoma; KIRP: Kidney papillary carcinoma; AML: Acute myeloid leukemia; OV: Ovarian cystadenocarcinoma; THCA: Thryroid carcinoma.

The mutational frequency of the 37 driver CRFs varies across cancer types (Figure 1A) –a behavior already observed in a smaller cohort [8]. While some CRFs (e.g., *MLL3*, *MLL2*) appear mutated in several cancer types, others are very specific to one or a few tumor types (e.g., *ATRX*, *PBRM1*), as apparent from differences in the distribution of the overrepresentation of CRF driver mutations (with respect to the expected value) in each tumor sample across all the cohorts analyzed, a metric which we call the CRF-to-driver index, or CDI (Methods). We computed the CDI as the minus logarithm of the pvalue of the Fisher's test of the overrepresentation of mutations in CRFs in each sample. While the CDI varies amongst the samples of each tumor type (Figure 1B) the median of its distribution in some cancer types –such as bladder carcinoma (BLCA) and cervix squamous cancer (CESC)–, is higher than in others. This result suggests that CRFs are involved more frequently in tumorigenesis in the cancer types of the former group.

### The CRFs-ODA identifies oncomodules related to *MLL2* driver mutations

**Figure 2:**
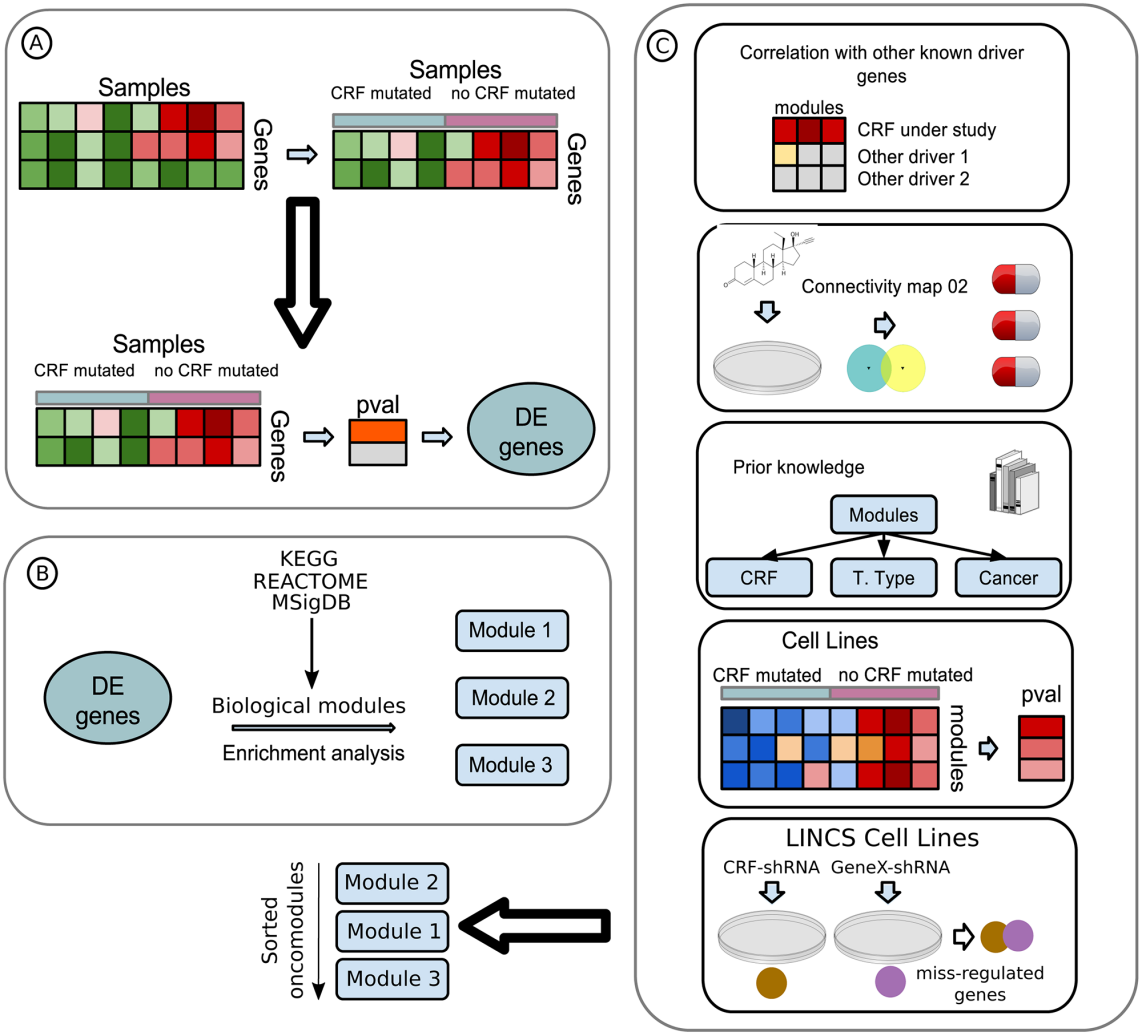
Flow diagram of the CRFs-ODA **A.** A data matrix with samples as columns and genes as rows is used as input. The genes (30%) with the lowest variance are discarded. Then, samples are separated following the mutational state of the driver CRF under study (details in Methods). The expression change between the two groups of samples of the remaining genes is computed, and those with corrected p-values below threshold are considered differentially expressed (DE). **B.** DE genes are analyzed for enrichment for several genesets, such as transcription factor targets from Transfac, biological pathways from KEGG and Reactome and experimentally generated oncomodules from MSigDB. Genesets with significant overrepresentation of DE genes (oncomodules) are retained for analysis. **C.** Oncomodules are sorted according to several layers of information obtained from the literature and cancer genomics and perturbaomics databases (Methods), in a process we refer to as a scoring system.

The three-step CRFs-ODA (Figure 2) is predicated on the idea that driver mutations in CRFs cause the miss-regulation of a set of functionally related downstream genes. First, the CRFs-ODA identifies genes whose expression changes significantly in tumors bearing driver mutations of a CRF with respect to unmutated samples (Figure 2A). Then, the CRFs-ODA identifies sets of functionally related genes (members of a biochemical pathway, with a common Gene Ontology term, or under the regulation of the same transcription factor) that are significantly enriched for the previously detected differentially expressed genes (Figure 2B). We call these sets oncomodules. Finally (Figure 2C), the CRFs-ODA employs a scoring system based on prior knowledge of the tumorigenesis across several cancer types to a) rank the biological modules detected in the previous step; b) detect spurious relationships between somatic alterations in the CRF and the differentially expressed genes; and c) devise hypotheses to explain how the CRF in question relates to the tumorigenic process and propose therapeutic strategies to target them. In this section, and the following two, we describe the use of the CRFs-ODA, illustrated through the detection of oncomodules in head and neck squamous cell carcinoma (HNSC) tumors carrying *MLL2* driver mutations Tables 1 and 2, and Supplementary Figure S1. We then summarize the results of its application to detect oncomodules related to mutations of CRFs in eleven cohorts of tumor samples analyzed by TCGA [9] (Supplementary Tables S1–S5).

**Table 1:** *MLL2* oncomodules detected in HNSC

Oncomodule	Query size	Term size	Overlap size	Adj. Pval
*SF1*	154	140	10	0.0019
mTOR	154	128	8	0.0118
*E2F1*	154	122	7	0.0247

**Query size:** Number of Differentially Expressed genes

**Term size:** Number of elements in the probed biological module

**Overlap size:** Number of elemens in the overlap between the set of differentially expressed genes and the sets of genes that form the probed biological module

**Adj. Pval:** P-value of the overrepresentation test correct for multiple testing

**Table 2:** Top-scoring oncomodules detected across all tumor cohorts

Tumor type	Driver CRFs	Top-scoring module	Correlation with other driver	CM02 drug modules	Prior CRF relation	Prior tumor type specific relation	Prior cancer relation	Miss-regulation in cancer cell lines	Overlap miss-regulation CRF/module	Overall score
HNSC	*MLL2*	mTOR	No	rapamycin, vorinostat	No	Yes	Yes	NA	Yes	5/6
		*E2F1*	No	No	Yes	Yes	Yes	NA	Yes	5/6
HNSC	*NSD1*	*MEK*	No	pioglitazone	Yes	Yes	Yes	NA	NA	5/5
		*AKT1*	No	trichostatin A, pioglitazone, LY-294002, rapamycin	No	Yes	Yes	NA	Yes	5/6
LUAD	*SMARCA4*	*SOX9*	No	estradiol	No	Yes	Yes	Yes	NA	5/6
		*HSF*	No	monorden (radicicol), estradiol, 15-dpj2, rapamycin	Yes	Yes	Yes	No	NA	5/6
KIRC	*PBRM1*	p53	No	LY-294002	Yes	Yes	Yes	No	Yes	6/7
		*ERBB2*	No	LY-294002	No	No	Yes	No	Yes	4/7
KIRC	*BAP1*	Base excision repair	No	vorinostat	Yes	Yes	Yes	No	NA	5/6
		CD 28 co-stimulation	No	trichostatin A, geldanamycin	No	Yes	Yes	No	NA	4/6
UCEC	*ARID1A*	p53	Yes (p53)	No	Yes	Yes	Yes	No	NA	NA
		Cell-cell junction	Yes (p53)	raloxifene, mefloquine	No	Yes	Yes	No	NA	NA

**Tumor type:** The tumor types names follow the same acronyms as in Figure 1.

**Driver CRFs:** Driver CRFs investigated with the CRFs-ODA in each tumor type.

**Top-scoring module:** Selected oncomodule(s), with the highest score for their misregulation upon mutations of driver CRFs in each tumor type.

**Correlation with other driver:** Miss-regulation of the oncomodule correlates with mutations of other driver better that with the CRF.

**CM02 drug modules:** Modules miss-regulated in response to drug perturbations that significantly (anti-)correlate with oncomodules, according to Connectivity Map 02. Drug names appear in each case.

**Prior CRF relation:** Evidences of the relationship between alterations of the CRF and miss-regulation of the oncomodule exist in the literature.

**Prior tumor type specific relation:** Evidences of the relationship between miss-regulation of the oncomodule and the emergence of this tumor type exist in the literature.

**Prior cancer relation:** Evidences of the relationship between miss-regulation of the oncomodule and tumorigenesis exist in the literature.

**Miss-regulation in cancer cell lines:** The oncomodule appears significantly miss-regulated in cancer cell lines bearing mutations of the CRF with respect to others without mutations of any CRF.

**Overlap miss-regulation CRF/module:** A significant overlap exists in genes miss-regulated upon knock-down of the CRF and knock-down of the gene controlling the oncomodule in cell lines.

**Overall score:** Fraction of the tests that support the involvement of the oncomodule in tumorigenesis upon mutations of the CRF.

To carry out the first step of the CRFs-ODA (Figure 2A), we retrieved the mutations and expression data of HNSC samples and divided them into two groups. The first group contained samples (N=52) bearing mutations of *MLL2* (all protein affecting mutations), while the second comprised the samples with no mutations in any driver CRF (N=60). To minimize the effects of the multiple test correction derived from the comparison of gene expression between the two groups, we discarded the 30% of genes with the smallest expression variance across samples. We then compared the expression of the remaining genes in the two groups of samples, using a Wilcoxon test followed by a Benjamini Hochberg FDR correction. We identified 154 differentially expressed (DE) genes −84 up-regulated and 70 down-regulated– (corrected P-value<0.05).

In the second step of the CRFs-ODA, we (Figure 2B), identified sets of functionally related genes (transcription factor targets from TRANSFAC [18], biochemical pathways from KEGG [19] and REACTOME [20] and oncogenic modules from MsigDB [21, 22]) significantly enriched for the DE genes. The 154 DE genes in HNSC were significantly enriched (Table 1) for genes of the mTOR pathway and for targets of the transcription factors *E2F1* and *SF1*. We refer to these genesets as the *MLL2* oncomodules in HNSC.

### A scoring system to rank oncomodules

We then ranked these three *MLL2* oncomodules using information retrieved from several cancer genomics and perturbaomics databases and the literature (Figure 2C) to implement the third step of the CRFs-ODA. First, we assessed whether mutations of any HNSC driver other than *MLL2* correlated better with the collective expression shift of the genes in each oncomodule than those of *MLL2*. To do this, we collapsed the expression values of the genes in each oncomodule in each sample into a Zscore value reflecting the level of collective over or under-expression of the module with respect to the population of all genes probed in the sample, through a Sample-Level Enrichment Analysis (SLEA: [23] and Methods). We then separated up-regulated and down-regulated DE genes within each oncomodule to compute their SLEA, thus producing a Zscore matrix of eight rows (six from the genes in the oncomodules and two for the whole sets of up- and down-regulated DE genes), as presented in Supplementary Figure S1A. Next, we compared the Zscores of samples grouped according to the mutations of each HNSC driver. We found that the mutational status of *MLL2* correlated better (Wilcoxon p-value smaller by more than 5 orders of magnitude; Supplementary Figure S1B) with the miss-regulation of the modules identified (mTOR, *E2F1* and *SF1*) than that of any other HNSC driver.

Secondly, we used the data in the Connectivity Map 02 [11], to search for gene signatures of response to therapeutic perturbations of cell lines that significantly correlate with the set of DE genes. The 154 differentially expressed genes identified in HNSC tumors upon mutations of *MLL2* showed a significant negative correlation with genes miss-regulated in several cell lines upon treatment with the mTOR inhibitor rapamycin, as well as with vorinostat, trichostatin A and LY-294002 and a positive correlation with genes miss-regulated in response to diethylstilbestrol (top 5 results; Supplementary Table S6).

As a third line of evidence to support and/or rank the detected oncomodules, we manually searched the literature for prior reports on the involvement of each CRF oncomodule in cancer. Specifically, we asked whether the miss-regulation of each oncomdule has previously been associated to: a) the activity of the CRF under study; b) the onset of tumorigenesis in the cancer type under analysis and/or; c) the onset of tumorigenesis in other tumor types. The three *MLL2* oncomodules have previously been linked to cancer [24–26], with *MTOR* and *E2F1* specifically involved in tumorigenesis in HNSC [27, 28]. Mutations of *MLL2* [29] have also been associated to the miss-regulation of *E2F1*.

As a fourth test, we asked whether the genes within each oncomodule are also miss-regulated in cancer cell lines with mutations of the CRF under study. To do this, we selected from the Cancer Cell Line Encyclopedia (CCLE) [10] all cell lines derived from tumors of the same cell type as the tumor type under analysis. Then, the cell line-wise Zscores of CRF oncomodules –representing the level of collective up- or down-regulation of each CRF oncomodule– were computed using the SLEA approach. Finally, the Zscores of cell lines bearing mutations of the CRF were compared to those of cell lines with the CRF unmutated. In the case of *MLL2*, because no information is available of the mutational status of *MLL2* in the CCLE, this test could not be performed. (See results for other CRFs in Supplementary Tables S1–S5.)

For the fifth and final test, we asked whether the significant overlap between DE genes upon mutations of the CRF and the set of genes within an oncomdule under the control of a gene (e.g. under the regulation of *E2F1*) was also observed in cell lines subjected to analogous perturbations. We computed the overlap between the set of genes most extremely miss-regulated in cell lines after knock-down of the CRF (in experiments carried out by the Library of Integrated Network-based Cellular Signatures, LINCS, http://www.lincsproject.org) and those extremely miss-regulated upon knock-down of the gene controlling the oncomodule. Miss-regulated genes upon knock-down of *MLL2* exhibit a significant overlap with those miss-regulated by perturbing the cell lines via loss-of-function of *MTOR*, *E2F1* and *SF1* (P-values, 3.9×10^−55^, 7.5×10^−58^, and 3.5×10^−23^, respectively).

In summary, the majority of the tests in the scoring system corroborate that mTOR, *E2F1* and *SF1* oncomodules constitute good candidates to mediate the tumorigenic effects of driver mutations of *MLL2* (see Discussion).

### Further evidences of the involvement of the mTOR oncomodule in *MLL2* mutated tumors

**Figure 3:**
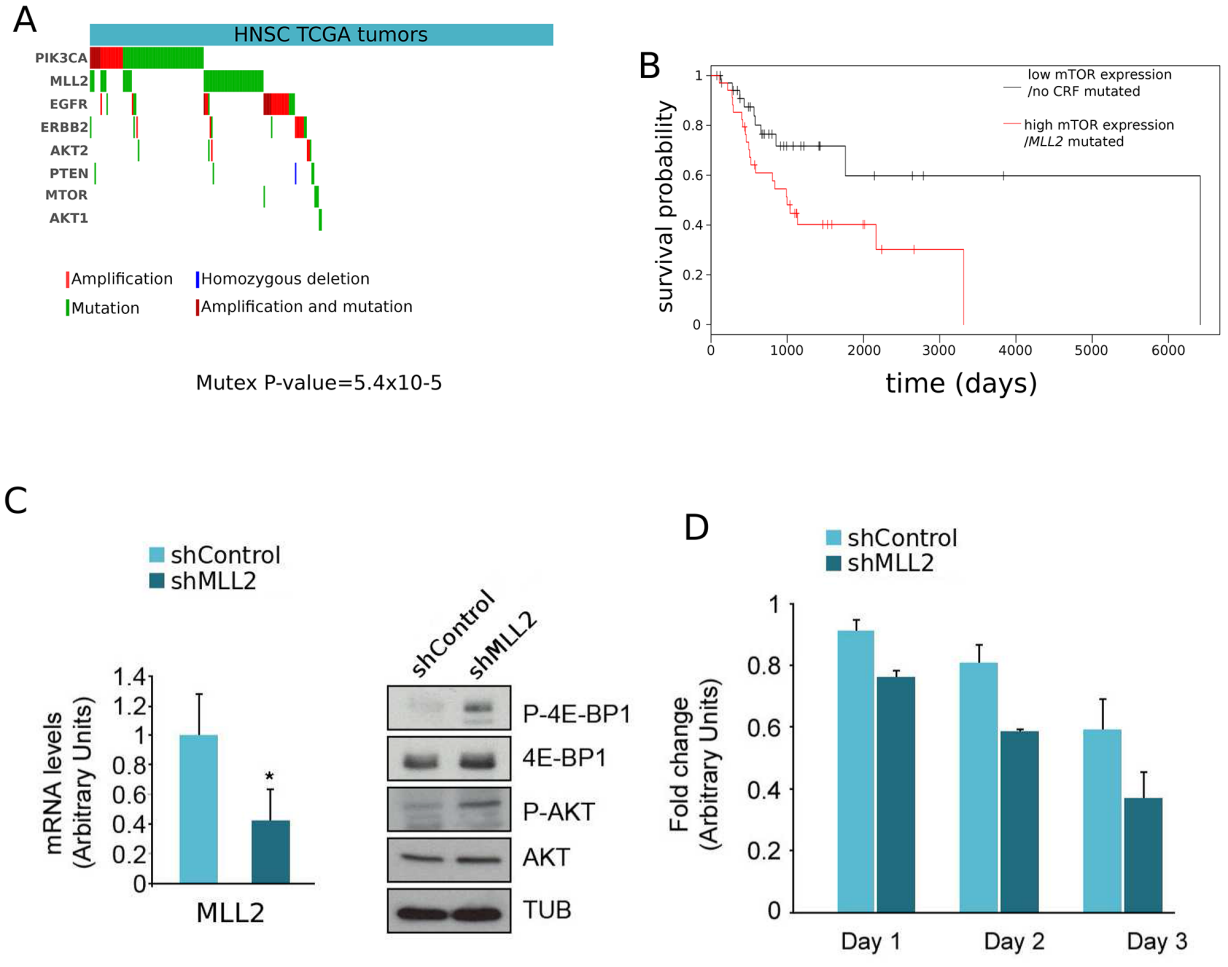
Further evidences supporting the involvement of mTOR in tumorigenesis upon mutations of *MLL2.* **A.** Mutual exclusivity of driver alterations of MLL2 and genes upstream and in the mTOR pathway. (Mutex p-value: 5.4×10^−5^) **B.** Loss-of-function mutations of MLL2 concomitant with miss-regulation of its related DE genes possess predictive survival value. HNSC tumors were separated in two groups: those bearing mutations of MLL2 and concomitant miss-regulation of related down-regulated genes (red curve), and those without mutations of MLL2 and no sign of down-regulation of the same genes. (A) Left panel. The levels of *MLL2* of lysates of T24 cells infected with an irrelevant short hairpin RNA (shControl) or specific for *MLL2* (shMLL2) were checked by real-time quantitative RT-PCR (qRT-PCR). Gene expression was normalized against an endogenous control and represented as RNA levels relative to those obtained in shControl-infected cells, which was set to 1. Right panel. The lysates were analysed by western blot with an anti-P-4E-BP1, 4E-BP1, P-AKT, AKT and Tubulin antibodies. (B) Knock-down of *MLL2* increased T24 cells sensitivity to everolimus treatment. The proliferation of both shControl and sh*MLL2* cells treated with everolimus in the course of 3 days (three replicates in each point) is presented relative to the proliferation of shControl and shMLL2 untreated cells, respectively. The units in the abscissa represent a proliferation ‘fold change’.

We determined that driver mutations of *MLL2* and alterations of driver genes upstream the mTOR pathway in HNSC occur in mutual exclusivity (P-value=5.4×10^−5^; Figure 3A), suggesting that all of them result in the same downstream alteration of the mTOR oncomodule. We also found that patients with *MLL2*-mutated HNSC tumors with high expression of the 84 genes that are significantly up-regulated upon such mutations exhibit significantly worst survival than patients with tumors with low expression of these same genes and no mutations in any CRF (Figure 3B). This constitutes an indication that the signatures of miss-regulated genes associated to driver mutations of CRFs may also carry prognostic value.

On the basis of all prior observations, we hypothesized that *MLL2* knockdown of a cancer cell line derived from a tumor type where *MLL2* drives cancerogenesis should produce the same type of miss-regulation of the mTOR oncomodule observed in head and neck primary tumors. Therefore, to simulate the downstream effects of loss of function mutations in *MLL2* and to investigate their relationships with alterations of the mTOR pathway, we carried out *MLL2* silencing with a specific short hairpin RNA in T24 human bladder cancer cells where the *MTOR* gene is not altered (Figure 3C, left panel). (We know that bladder carcinoma is one of the tumor types frequently driven by *MLL2* loss-of-function mutations: see http://www.intogen.org/search?gene=MLL2&cancer=BLCA). Since mTORC1 activity is required for 4E-BP1 phosphorylation and mTORC2 for AKT phosphorylation, we analyzed the phosphorylation status of these two proteins in the absence of *MLL2* by western blot. In agreement with the predictions resulting from our scoring system, the decrease in *MLL2* expression, checked by quantitative PCR, resulted in increased mTORC1/2 activity (Figure 3C, right panel), which in turn suggests that these cancer cells may be more sensitive to mTOR pathway inhibitors. To test this hypothesis, we treated T24 cells, with and without the *MLL2* shRNA insertion, with everolimus and measured their growth rate through an MTT assay. As shown in Figure 3D, everolimus proved more effective in the inhibition of the growth of cells carrying the *MLL2* shRNA.

### Potential mechanisms of tumorigenesis of other driver CRFs

We identified oncomodules associated to the alterations of six CRFs in four cancer types (including *MLL2* in HNSC). The results of the analyses are summarized in Table 2 and presented at length in Supplementary Tables S1 to S5. For example, while mTOR and *E2F1* are the top-ranking oncomodules associated to mutations of *MLL2* in HNSC, we found that oncomodules in the *MEK*/*AKT1* axis are top-ranking in association to *NSD1* mutations in the same cancer type. In the case of mutations of *SMARCA4* in lung adenocarcinomas (LUAD), the top ranking oncomodules include SOX-9 and transcription factors of the HSF family, which have been linked to tumorigenesis before ([30]; Supplementary Table S2). In kidney clear cell carcinomas (KIRC) the top-ranking oncomodule associated to mutations of *PBRM1* (the most frequently mutated KIRC driver, [31] are genes in the p53 pathway (Supplementary Table S3). On the other hand, genes related to base-excision repair mechanisms constitute the top-ranking oncomodule related to driver mutations of *BAP1*, another frequently mutated CRF in KIRC (Supplementary Table S4). While genes within the p53 pathway are significantly enriched for DE genes in uterine endometriod carcinomas (UCEC) bearing mutations of *ARID1A* and unmutated ones, collective differences in expression of genes in the pathway correlate more significantly with driver mutations of *TP53* (Supplementary Table S5). Mutations of *TP53* thus constitute a much simpler explanation of the observed miss-regulation of genes under its control, and we accept it under Occam's razor.

## DISCUSSION

We developed a CRFs-ODA to prioritize sets of functionally related genes miss-regulated upon somatic mutations of driver CRFs (oncomodules). We applied it to 11 cohorts of tumors analyzed by TCGA, and identified top-ranking oncomodules associated to 5 CRFs in 3 cancer types. To our knowledge, this constitutes the first systematic analysis of the oncomodules that become miss-regulated upon mutations of driver CRFs across cancer types. We focused on the top-ranking oncomodules associated to mutations of *MLL2* in HNSC to illustrate the validity of our approach, and we made predictions on how the perturbation of the oncomodules could render the tumors sensitive to certain anti-cancer drugs. Using the Connectivity Map 02, for instance, we found that drugs inhibiting mTOR (Rapamycin) and histone de-acetylases (HDAC inhibitors) could constitute candidates to indirectly target *MLL2*-deficient tumors. Previous studies have shown that Vorinostat enhances the ability of mTOR inhibitors to induce cell death [32]. We also made other observations that support the mechanistic relationship between the loss of function of *MLL2* and the miss-regulation of genes in the mTOR pathway in tumorigenesis, such as the mutual exclusivity of mutations across them. In addition, we experimentally observed that –as predicted by this hypothetic mechanistic relationship– the loss of function of *MLL2* in cell lines derived from tissues in which *MLL2* drives tumorigenesis renders tumor cells more sensitive to mTOR inhibitors. Note that neither the *in silico* predictions resulting from our scoring system nor the experimental results that back them are able to demonstrate the existence of a direct link between *MLL2* and the mTOR pathway. Our results could also be due to synthetic lethality. Rather than as an experimental validation of this particular link bettwen loss-of-function mutations of *MLL2* and the miss-regulation of the *MTOR* module –which, outside the scope of our study, must be undertaken by the cancer research community– this result lends support to the validity of our approach.

Description of the oncogenic modules related to five CRFs in three tumor types with biologically meaningful results, together with all the information produced by the CRFs-ODA and –in particular– the scoring system on each of them are available to cancer genomics researchers as Supplementary Tables S1–S5. These results constitute a pool of hypotheses on the mechanisms through which *MLL2*, *NSD1*, *SMARCA4*, *PBRM1*, and *BAP1* may trigger the malignization of cells in HNSC, LUAD, and KIRC. We envision that these hypotheses be tested experimentally, and in particular that indirect therapeutic strategies proposed by the strategy be essayed for their potential use in clinical settings. We also envision that the strategy we have developed in this study be used to explore the tumorigenic mechanisms of other CRFs –and eventually other driver– as larger multidimensional cancer genomics datasets become available from new and bigger sequencing studies.

## MATERIALS AND METHODS

### Data download and processing

Mutations in driver genes in 6792 tumors from 29 cancer types to carry out the mutational landscape analysis, were downloaded from IntOGen [1, 33]. (We added to the mutational frequency analysis a cohort of lung tumors of unknown histology. See Figure 1A.) Both the expression data and the mutational information used in the differential expression analysis were retrieved from TCGA through the import capability of the Gitools program [34]. Expression data for 3583 tumor samples form 12 different cancer types was already normalized and median-centered. The sets of functionally related genes used in enrichment analyses (see below) were downloaded from MsigDB [21, 22]. They encompassed TRANSFAC transcription factor targets, KEGG and REACTOME biological pathways and experimental oncogenic signatures. The Cell lines expression and mutational data used in the scoring section was downloaded from the Cancer Cell Line Encyclopedia [10]. Genes in cell lines with knocked-down CRFs and other genes were obtained from the Library of Integrated Network-based Cellular Signatures (LINCS; http://www.lincsproject.org/) program.

### Differential expression analysis

The variance of every gene across all the samples available, regardless of their mutational status was computed. The 30% of the genes with lowest variance were discarded. Next, the samples were divided into two groups, one group contained the samples with protein affecting mutations (PAMs) in the CRF under study, while the other was composed of the samples with no mutations of driver CRFs. Then, a Wilcoxon test comparing the expression of every gene between these two groups was performed. The resulting p-values were subjected to multiple test correction using the Benjamini Hochberg FDR method. The expression comparison, p-value correction and filtering was carried out using Gitools [34]. Finally, genes with an adjusted p-value lower than 0.05 were considered as differentially expressed. When the number of differentially expressed genes was higher than 1000, the adjusted p-value threshold was raised to 0.01. If the resulting list of differentially expressed genes had still more than 1000 genes the adjusted p-value threshold was raised again to 0.001.

### Enrichment analysis

Hypergeometric tests followed by the corresponding multiple test correction were performed between the differentially expressed genes and every one of the sets of genes of functionally related genes mentioned in the first section. Tests with an adjusted p-value lower than 0.05 were considered statistically significant.

### Construction of oncogenic modules and sample level enrichment analysis

We constructed oncogenic modules, i.e., sets of genes differentially expressed in coherence with the occurrence of driver mutations in the CRF under analysis and related with cellular functions. Each oncogenic module contained differentially expressed genes overlapping sets of genes that were significant in the previously described enrichment analysis. Genes in these sets which exhibited raw p-values lower than 0.05 in the differential expression analysis, but discarded due to the multiple test correction, were added back to the oncogenic module. Each oncogenic module inherited its name from the original gene set which significantly overlapped the differentially expressed genes. Next, every oncogenic module was divided into two subsets of genes, one containing the up-regulated genes, and the second one, with the down-regulated genes. The resulting subsets of genes were used as input for the Sample Level Enrichment Analysis (SLEA; [23]) implemented in Gitools [34].

### Correlation of the miss-regulation of oncogenic modules with other drivers

A list of cancer driver genes mutated in 5 or more samples from the differential expression analysis was retrieved. Next, a SLEA using as input the expression data used in the differential expression analysis and as gene sets those built as explained in the SLEA section was performed. Every one of the genes in the list of cancer driver genes with more than 5 mutations mentioned above was used to group the samples according to its mutational status, then performing a group comparison of the Z scores resulting from the SLEA. Thus, a p-value per cancer driver gene per gene set was obtained. Finally, these p-values were ranked to check whether the most significant p-value corresponded to the CRF under study. If so, the modules received a positive score.

### Miss-regulation of oncomodules in cancer cell lines

Cell lines data corresponding to cell lines derived from the same tissue than the tumor type under study were selected. Cell lines expression data was used to perform a SLEA with the gene sets built as described in the SLEA section. Thus, a Z score per gene set per cell line was obtained. Next, cell lines were grouped according to the mutational status of the CRF under study and Z-scores were compared using a Wilcoxon test between the two groups, followed by a multiple test correction (Benjamini-Hochberg FDR method). Modules whose Z scores group comparison appeared to be statistically significant received a positive score.

### Overlap of genes miss-regulated upon knock-down of CRFs and oncomodules in cell lines

We analyzed the overlap between the genes that become miss-regulated when a CRF is knocked-down with those miss-regulated when the gene controlling each of the oncomodules detected to be associated with the CRF is knocked-down. Genes miss-regulated upon knock-down of a gene were obtained from the experiments carried out in cell lines by the LINCS program (see above). We require that the genes appear as extremely miss-regulated in at least two knock-down experiments to include them in the sets to test the significance of the overlap. We only carried out this test when the oncomodule in question was unequivocally under the control of a gene, rather than describing a biological process and at least one knock-down experiment had been carried out within LINCS of the CRF and the gene controling the module. We then probed the significance of the overlapping set of genes through a Fisher's test.

### Mining prior knowledge on detected oncomodules

An exhaustive literature search was performed in order to assess whether the modules identified in the enrichment analysis had already been related with mutations in the CRF under study, the tumor type or cancer in general. Each one of these already identified relations was scored positively in case of being reported on literature.

### Detecting correlation between oncomodules and perturbation-response genesets

To fulfill the input format requirements of the Connectivity Map 02, the genes identified as differentially expressed in the differential expression analysis were divided into two subsets, containing the up and down regulated genes respectively. Also, the gene ids of the genes in this two subsets were converted from symbol to probe id (when more than a probe belonged to the same gene, all of them were added). Next the two subsets of genes were used as input to run CM02. The top 5 resulting drugs were selected, but only if the number of instances was higher greater or equal to 5 (or in other words, that had been tested at least 5 times varying the concentration, the cell-line or the batch). If any of this drugs had as target one of the modules identified in the enrichment analysis or had been related to it, the module was scored positively.

### Mutual exclusivity test

We first manually selected genes within the *PIK3CA* pathway finalizing with signalling through *MTOR*. Then, to visualize and assess the significance of the mutual exclusivity of alterations of these genes, we once again employed Gitools [34] built-in capabilities. After automatically sorting the genes following the mutually exclusive pattern of their alterations, we carried out the Mutex test implemented in Gitools which permutes the alterations observed in each gene in the set across the samples in the cohort respecting the observed probability of alterations in each of them. Then, it assesses the likelihood that the number of samples affected by the observed pattern of alterations appeared by chance by comparing it to those resulting from the permutations and computing an empirical P-value.

### Testing the sensitivity of *MLL2*-knocked down cells to everolimus

#### Compounds

Everolimus was purchased from Sigma-Aldrich.

#### Cell culture

Human T24 cell line was obtained from the American Type Culture Collection (Manassas, VA, USA). Cells were maintained in DMEM medium supplemented with 10% heat-inactivated fetal bovine serum, penicillin (100 IU ml^−1^), streptomycin (100 mg×ml^−1^) and 4 mM glutamine (ICN, Irvine, UK) in a humified atmosphere of 95% air and 5% CO_2_ at 37°C. For lentiviral infection, HEK293T cells were used to produce viral particles. Cells were transfected (day 0) by adding drop-wise NaCl together with a DNA mixture comprising 50% pLKO-shControl/shKMT2D (Mission library Sigma SHCLNG-NM_003482), 10% pCMV-VSVG, 30% pMDLg/pRRE and 10% pRSV rev and polyethylenimine polymer (Polysciences Inc) that were preincubated for 15 min at room temperature. The transfection medium was replaced with fresh medium after 24 h (day 1), and the cell-conditioned medium at day 2 was filtered and used to infect target cells with 8 μg/mL polybrene. HEK293T cells were incubated with fresh medium for further 24 h, and a second infection with the conditioned medium and polybrene was performed on day 3. Infected cells were selected with puromycin for 72 h (2 μg/mL).

### RNA analysis by quantitative RT-PCR (qRT-PCR)

After RNA extraction with TRIzol® reagent (Invitrogen), RNA was retrotranscribed with the transcription first-strand cDNA synthesis kit (Roche), and real-time quantitative PCR experiments were done in a Light Cycler PCR machine (Roche). This was used to verify the efficiency of the *MLL2* KD.

### Cell survival assay

Cells (5×10^4^ cells per well) were grown in 24-well plates and exposed to 100nM of the drug. The percentage of cell growth was determined using the 3-(4,5-dimethylthiazol-2-yl)-2,5-diphenyltetrazolium bro- mide (MTT) assay according to the manufacturer's instructions.

### Western blot analysis

Total cell lysates were obtained from cell cultures. Protein extracts were resolved by 10% SDS–PAGE and probed with anti-human, polyclonal P-Akt Thr308 (#9275, Cell Signaling), Akt (#9272, Cell Signaling), P-4E-BP1 Thr37/46 (#9459, Cell Signaling), 4E-BP1 (#9452, Cell Signaling) and Tubulin (T9026, Sigma) antibodies. Immunoreactive proteins were visualised by enhanced chemiluminescence (Pierce, Rockford, IL, USA).

## SUPPLEMENTARY FIGURE AND TABLES














