# Supplementary material for: Altered oncomodules underlie chromatin regulatory factors driver mutations
Source: Oncotarget. 2016 Apr 15;7(21):30748–59. doi: 10.18632/oncotarget.8752 (PMC5058714; doi:10.18632/oncotarget.8752)
Supplement: Supplementary file 1 [file oncotarget-07-30748-s001.pdf]

Altered oncomodules underlie chromatin regulatory factors driver mutations

SUPPLEMENTARY FIGURE AND TABLES

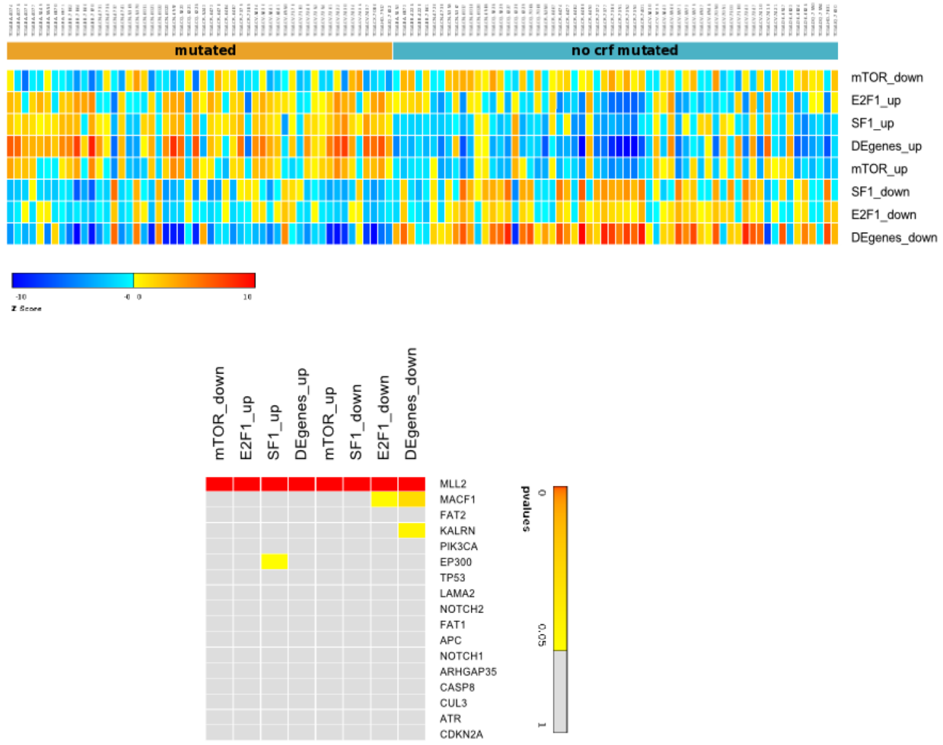

Supplementary Figure S1: MLL2 oncomodules detected by the CRFs Oncomodules Discovery approach in HNSC. Top panel. Correlation of the miss-regulation of Oncomodules (measured using SLEA) with driver MLL2 loss-of-function mutations. **Bottom panel.** Correlation of the miss-regulation of Oncomodules with mutations of other HNSC drivers. Cells in the heatmap represent the p-values of Mann-Whitney comparison of SLEA Zscore values.

Supplementary Table S1: Top ranking Oncomodules of the CRFs Oncomodules Discovery associated to driver mutations of NSD1 in HNSC.

See Supplementary File 1

Supplementary Table S2: Top ranking Oncomodules of the CRFs Oncomodules Discovery associated to driver mutations of SMARCA4 in LUAD.

See Supplementary File 2

Supplementary Table S3: Top ranking Oncomodules of the CRFs Oncomodules Discovery associated to driver mutations of PBRM1 in KIRC.

See Supplementary File 3

Supplementary Table S4: Top ranking Oncomodules of the CRFs Oncomodules Discovery associated to driver mutations of BAP1 in KIRC.

See Supplementary File 4

Supplementary Table S5: Top ranking Oncomodules of the CRFs Oncomodules Discovery associated to driver mutations of ARID1A in UCEC.

See Supplementary File 5

Supplementary Table S6: Top ranking drugs targeting the Oncomodules associated to MLL2 in HNSC according to the Connectivity Map Cmap (Connectivity Map).

See Supplementary File 6
