# Supplementary material for: Altered oncomodules underlie chromatin regulatory factors driver mutations
Source: Oncotarget. 2016 Apr 15;7(21):30748–59. doi: 10.18632/oncotarget.8752 (PMC5058714; doi:10.18632/oncotarget.8752)
Supplement: Supplementary file 2 [file oncotarget-07-30748-s002.docx]

**Supplemental table 1. Top ranking Oncomodules of the CRFs Oncomodules Discovery associated to driver mutations of NSD1 in HNSC**

| **NSD1 in HNSC (Head and Neck Squamous Cell Carcinoma)** | | | | | | | | | | | | |
| --- | --- | --- | --- | --- | --- | --- | --- | --- | --- | --- | --- | --- |
| Samples mutated | Samples no CRF mutated | Adjusted P-value threshold | DE genes | Top 5 Connectivity Map 02 drugs identified | Modules identified | Other drivers | CM02 drugs | Prior knowledge CRF | Prior knowledge tumor type | Prior knowledge cancer | Significant in CCLE | Overlap miss-regulated genes CRF/module |
| 31 | 60 | 0.01 | 725 | +pioglitazone  -LY-294002  -tanespimycin  -trichostatin A  -sirolimus | AKT1 | No | Yes (trichostatin A, pioglitazone, LY-294002, rapamycin) | No | Yes | Yes | NA | Yes |
|  |  |  |  |  | Late serum response of CRL | No | No | No | No | No | NA | NA |
|  |  |  |  |  | ATF2 | No | No | No | Yes | Yes | NA | Yes |
|  |  |  |  |  | TGFB | No | Yes (richostatin A, pioglitazone, rapamycin) | No | Yes | Yes | NA | NA |
|  |  |  |  |  | LTE2 (long-term adapted for estrogen-independent growth) | No | No | Yes | No | Yes | NA | NA |
|  |  |  |  |  | Metabolism of carbohydrates | No | Yes (pioglitazon) | No | Yes | Yes | NA | NA |
|  |  |  |  |  | Glycosphingolipid biosynthesis lacto and neolacto series | No | Yes (rapamycin) | No | Yes | Yes | NA | NA |
|  |  |  |  |  | E2F1 | No | No | No | Yes | Yes | NA | Yes |
|  |  |  |  |  | mTOR | No | Yes (rapamycin) | No | Yes | Yes | NA | Yes |
|  |  |  |  |  | MEK | No | Yes (pioglitazone) | Yes | Yes | Yes | NA | NA |
|  |  |  |  |  | knockdown of BMI1 and PCGF2 | No | No | No | Yes | Yes | NA | NA |
|  |  |  |  |  | Genes up-regulated in oligodendrocytes. | No | Yes (pioglitazone, LY- 294002) | No | No | No | NA | NA |

**Samples mutated:** Number of tumor samples with mutations of the CRF in the cohort

**Samples no CRF mutated:** Number of tumor samples without mutations of any CRF

**Adjusted P-value threshold:** Threshold of adjusted p-value to consider a gene as differentially expressed

**DE genes:** Number of genes with expression difference adjusted p-value below the threshold

**Modules identified:** Genesets identified as significantly enriched for DE genes; i.e., potential oncomodules miss-regulated upon driver alterations of the CRF

**Other drivers:** Existence of a correlation (Mann-Whitney comparison of SLEA Zscores) of the miss-regulation of oncomodules with alterations of other drivers of the tumor type higher than that observed with alterations of the CRF

**CM02 drugs:** Drugs which perturb the expression of genesets that significantly (measured through connectivity map 02 correlations) overlap oncomodules

**Prior knowledge CRF:** Previous reports exist associating the miss-regulation of the identified oncomodule with alterations of the CRF

**Prior knowledge tumor type:** Previous reports exist associating the miss-regulation of the oncomodule with cancerogenesis in the tumor type of the cohort

**Prior knowledge cancer:** Previous reports exist associating the miss-regulation of the oncomodule with cancerogenesis in some tumor types

**Significant in CCLE**: Collective miss-regulation of the genes in the oncomodule in cancer cell lines derived from the same tissue of origin as the cohort significantly correlate (Mann-Whitney comparison of SLEA Zscores) with alterations of the CRF in the same cell lines.

**Overlap miss-regulated genes CRF/module:** Sets of genes extremelly miss-regulated upon knock-down of the CRF (in experiments carried out in cell lines by LINCS) significantly overlap sets of genes extremely miss-regulated upon knock-down of the gene controlling the oncomodule (in cases where the latter can unequivocally be discerned).
