# Supplementary material for: Altered oncomodules underlie chromatin regulatory factors driver mutations
Source: Oncotarget. 2016 Apr 15;7(21):30748–59. doi: 10.18632/oncotarget.8752 (PMC5058714; doi:10.18632/oncotarget.8752)
Supplement: Supplementary file 3 [file oncotarget-07-30748-s003.docx]

**Supplemental table 2. Top ranking Oncomodules of the CRFs Oncomodules Discovery associated to driver mutations of SMARCA4 in LUAD**

| **SMARCA4 in LUAD (Lung Adenocarcinoma)** | | | | | | | | | | | | |
| --- | --- | --- | --- | --- | --- | --- | --- | --- | --- | --- | --- | --- |
| Samples mutated | Samples no CRF mutated | Adjusted P-value threshold | Number DE genes | Top 5 Connectivity Map 02 drugs identified | Modules identified | Better correlation with any other driver | Related with CM02 results | Previously related with the CRF | Previously related with the tumor type | Previously related with cancer | Significant in CCLE | Overlap miss-regulated genes CRF/module |
| 14 | 22 | 0.05 | 76 | +15-delta prostaglandin J2  +estradiol  +monorden  -rapamycin  +MG-262 | DBP | No | No | No | Yes | Yes | Yes | NA |
|  |  |  |  |  | SOX9 | No | Yes (estradiol) | No | Yes | Yes | Yes | NA |
|  |  |  |  |  | Metabolism of nucleotides | No | No | No | Yes | Yes | No | NA |
|  |  |  |  |  | HSF | No | Yes (monorden (radicicol), estradiol, 15-dpj2, rapamycin) | Yes | Yes | Yes | No | NA |
|  |  |  |  |  | STK33 | No | No | No | Yes | Yes | Yes | Yes |
|  |  |  |  |  | TEF1 | No | No | No | No | Yes | Yes | Yes |
