# Supplementary material for: Altered oncomodules underlie chromatin regulatory factors driver mutations
Source: Oncotarget. 2016 Apr 15;7(21):30748–59. doi: 10.18632/oncotarget.8752 (PMC5058714; doi:10.18632/oncotarget.8752)
Supplement: Supplementary file 4 [file oncotarget-07-30748-s004.docx]

**Supplemental table 3. Top ranking Oncomodules of the CRFs Oncomodules Discovery associated to driver mutations of PBRM1 in KIRC**

| **PBRM1 in KIRC (Kidney Renal Clear Cell Carcinoma)** | | | | | | | | | | | |  |
| --- | --- | --- | --- | --- | --- | --- | --- | --- | --- | --- | --- | --- |
| Samples mutated | Samples no CRF mutated | Adjusted P-value threshold | Number DE genes | Top 5 Connectivity Map 02 drugs identified | Modules identified | Better correlation with any other driver | Related with CM02 results | Previously related with the CRF | Previously related with the tumor type | Previously related with cancer | Significant in CCLE | Overlap miss-regulated genes CRF/module |
| 133 | 89 | 0.01 | 586 | +LY-294002 | LTE2 (long-term adapted for estrogen-independent growth) | No | No | No | No | Yes | No | NA |
|  |  |  |  |  | p53 | No | Yes (LY-294002) | Yes | Yes | Yes | No | Yes |
|  |  |  |  |  | ERBB2 | No | Yes (LY-294002) | No | No | Yes | No | Yes |
|  |  |  |  |  | Arginine and proline metabolism | No | No | No | No | No | No | NA |
|  |  |  |  |  | JANK MAPK | No | No | No | Yes | Yes | No | NA |
