# Supplementary material for: Altered oncomodules underlie chromatin regulatory factors driver mutations
Source: Oncotarget. 2016 Apr 15;7(21):30748–59. doi: 10.18632/oncotarget.8752 (PMC5058714; doi:10.18632/oncotarget.8752)
Supplement: Supplementary file 5 [file oncotarget-07-30748-s005.docx]

**Supplemental table 4. Top ranking Oncomodules of the CRFs Oncomodules Discovery associated to driver mutations of BAP1 in KIRC**

| **BAP1 in KIRC (Kidney Renal Clear Cell Carcinoma)** | | | | | | | | | | | |  |
| --- | --- | --- | --- | --- | --- | --- | --- | --- | --- | --- | --- | --- |
| Samples mutated | Samples no CRF mutated | Adjusted P-value threshold | Number DE genes | Top 5 Connectivity Map 02 drugs identified | Modules identified | Better correlation with any other driver | Related with CM02 results | Previously related with the CRF | Previously related with the tumor type | Previously related with cancer | Significant in CCLE | Overlap miss-regulated genes CRF/module |
| 39 | 89 | 0 | 788 | -trichostatin A  -vorinostat  -geldanamycin | Glycosylphosphatidylinositol gpi anchor biosystem | No | No | No | Yes | Yes | No | NA |
|  |  |  |  |  | Base excision repair | No | Yes (vorinostat) | Yes | Yes | Yes | No | NA |
|  |  |  |  |  | p75ntr | No | Yes (trichostatin A) | No | No | Yes | No | NA |
|  |  |  |  |  | CTLA4 inhibitory signal | - | - | - | - | - | - | NA |
|  |  |  |  |  | CD28 costimulation | No | Yes (trichostatin A, geldanamycin) | No | Yes | Yes | No | NA |
