# Supplementary material for: Altered oncomodules underlie chromatin regulatory factors driver mutations
Source: Oncotarget. 2016 Apr 15;7(21):30748–59. doi: 10.18632/oncotarget.8752 (PMC5058714; doi:10.18632/oncotarget.8752)
Supplement: Supplementary file 6 [file oncotarget-07-30748-s006.docx]

**Supplemental table 5. Top ranking Oncomodules of the CRFs Oncomodules Discovery associated to driver mutations of ARID1A in UCEC**

| **ARID1A in UCEC (Uterine Corpus Endometrioid Carcinoma)** | | | | | | | | | | | | |
| --- | --- | --- | --- | --- | --- | --- | --- | --- | --- | --- | --- | --- |
| Samples mutated | Samples no CRF mutated | Adjusted P-value threshold | Number DE genes | Top 5 Connectivity Map 02 drugs identified | Modules identified | Better correlation with any other driver | Related with CM02 results | Previously related with the CRF | Previously related with the tumor type | Previously related with cancer | Significant in CCLE | Overlap miss-regulated genes CRF/module |
| 65 | 53 | 0 | 421 | +raloxifene  -tridihexethyl  +PHA-00851261E  +mefloquine  -tobramycin | Interferon signaling | Yes (p53) | Yes (raloxifene) | No | No | Yes | No | NA |
|  |  |  |  |  | TAP63 | Yes (p53) | No | No | Yes | Yes | No | NA |
|  |  |  |  |  | Cell-cell junction organization | Yes (p53) | Yes (raloxifene, mefloquine) | No | Yes | Yes | No | NA |
|  |  |  |  |  | ERBB2 | Yes (p53) | No | No | Yes | Yes | No | NA |
|  |  |  |  |  | p53 | Yes (p53) | No | Yes | Yes | Yes | No | NA |
|  |  |  |  |  | mTOR | Yes (p53) | Yes (mefloquine) | No | Yes | Yes | No | NA |
