# Supplementary material for: Altered oncomodules underlie chromatin regulatory factors driver mutations
Source: Oncotarget. 2016 Apr 15;7(21):30748–59. doi: 10.18632/oncotarget.8752 (PMC5058714; doi:10.18632/oncotarget.8752)
Supplement: Supplementary file 7 [file oncotarget-07-30748-s007.docx]

**Supplemental table 6.** **Top ranking drugs targeting the Oncomodules associated to MLL2 in HNSC according to the Connectivity Map**

Cmap (Connectivity Map)

| **rank** | **Cmap name** | **mean** | **n** | **enrichment** | **p** | **specificity** | **Percent non-null** |
| --- | --- | --- | --- | --- | --- | --- | --- |
| 1 | vorinostat | -0.48 | 12 | -0.63 | 0 | 0.24 | 83 |
| 2 | Trichostatin A | -0.424 | 182 | -0.48 | 0 | 0.24 | 78 |
| 3 | LY-294002 | -0.314 | 61 | -0.41 | 0 | 0.14 | 63 |
| 4 | sirolimus | -0.398 | 44 | -0.4 | 0 | 0.19 | 77 |
| 5 | diethylstillbestrol | 0.551 | 6 | 0.74 | 0 | 0.04 | 83 |
